# Call for amendment of *Declaration of Geneva* of the World Medical Association

**DOI:** 10.4102/jcmsa.v2i1.12

**Published:** 2024-03-06

**Authors:** Johannes J. Fagan, Salome Maswime, Mark G. Shrime

**Affiliations:** 1 Division of Otolaryngology, Faculty of Health Sciences, University of Cape Town, Cape Town, South Africa; 2 Division of Global Surgery, Faculty of Health Sciences, University of Cape Town, Cape Town, South Africa; 3 Department of Global Health and Social Medicine, Faculty of Global Health and Social Medicine, Harvard Medical School, Boston, United States of America

**Keywords:** Declaration of Geneva, amendment, resource limitations, societal responsibility, triage

## Abstract

The *Declaration of Geneva* serves as a guide to ethical medical practice. It primarily addresses the duties of the physician in relation to an individual physician–patient relationship and implicitly advocates a ‘first come, first served’ model. It assumes the availability of adequate resources to treat all patients. However, no health system can meet all the requirements of its intended beneficiaries, and resource allocation, priority-setting and triaging are inevitable. Yet the *Declaration of Geneva* ‘does not permit considerations of age, disease or disability, gender …, social standing or any other factor’ to be considered. Neither does it permit consideration of ‘financial toxicity of treatment’ on patients, families and struggling healthcare systems. Making resource allocation, priority-setting, and triaging decisions is ethically complex. Yet in many resource-limited settings, such difficult and ethical judgement calls are left to individual physicians to make; this applies especially in low- and middle-income countries where practitioners are often faced with overwhelming burdens of disease and simply cannot treat everyone requiring care. The *Declaration of Geneva* should be amended to recognise limitations of physicians to deliver care because of health system constraints and should speak not only of a physician’s duty towards the individual patient but also to broader society. It should provide ethical guidance to those practising in limited resource settings about triaging, protecting elective care, ensuring training of well-rounded physicians, ensuring financial wellness of patients and healthcare systems and ensuring accountability for health and wellness of patients and healthcare systems.

## Introduction

Clinicians are taught to consider patients one-by-one and to do the best for the individual patient. Yet, no health system, whether public or private can meet all the requirements of its intended beneficiaries, and resource allocation and priority-setting are inevitable.^1^ Making such decisions is ethically complex.^1^ Yet in many resource-limited settings such difficult and ethical judgement calls are left to individual physicians to make, despite their training not preparing them to do so, and paucity of ethical guidance to resource allocation and priority setting.

The *Declaration of Geneva* serves as a guide to ethical medical practice (Box 1).^2^ It was introduced following unethical practice of physicians under the Nazi regime during World War II and builds on the principles contained in the *Hippocratic Oath*.^2^ It was first adopted in 1948 by the *World Medical Association* (WMA) and has subsequently been amended in 1968, 1983, 1994, 2005, 2006 and again in 2017.^2^

BOX 1 The physician’s pledge (*Declaration of Geneva*).

**AS A MEMBER OF THE MEDICAL PROFESSION:**

I SOLEMNLY PLEDGE to dedicate my life to the service of humanity;

THE HEALTH AND WELL-BEING OF MY PATIENT will be my first consideration;

I WILL RESPECT the autonomy and dignity of my patient;

I WILL MAINTAIN the utmost respect for human life;

I WILL NOT PERMIT considerations of age, disease or disability, creed, ethnic origin, gender, nationality, political affiliation, race, sexual orientation, social standing or any other factor to intervene between my duty and my patient;

I WILL RESPECT the secrets that are confided in me, even after the patient has died;

I WILL PRACTISE my profession with conscience and dignity and in accordance with good medical practice;

I WILL FOSTER the honour and noble traditions of the medical profession;

I WILL GIVE to my teachers, colleagues, and students the respect and gratitude that is their due;

I WILL SHARE my medical knowledge for the benefit of the patient and the advancement of healthcare;

I WILL ATTEND TO my own health, well-being, and abilities in order to provide care of the highest standard;

I WILL NOT USE my medical knowledge to violate human rights and civil liberties, even under threat;

I MAKE THESE PROMISES solemnly, freely, and upon my honour.

*Source:* World Medical Association, WMA Declaration of Geneva, [homepage on the Internet]. c2018 [cited 2024 Feb 01]. Available from: https://www.wma.net/policies-post/wma-declaration-of-geneva/

The principles enshrined in the *Declaration of Geneva* serve as a guide for clinicians and healthcare planners and regulators for ethical practice. Rheinsberg et al.^3^ reported in a members’ survey of the WMA, that even though the *Declaration of Geneva* is not widely used as an oath, it is frequently included in medical professional codes of conduct or used as a point of reference. The authors reported that in 23/37 (62%) countries surveyed, the *Declaration of Geneva* was not legally binding; in 6/37 (16%), it was legally binding in professional regulations or professional law, and in 2/37 (5%), it was legally binding under general law.^3^ Six membership organisations (16%) indicated that the *Declaration of Geneva* was not used in their countries and three (8%) did not respond. Not adhering to the principles contained in the *Declaration of Geneva* may therefore have legal consequences for healthcare practitioners and administrators.

## *Declaration of Geneva* and limited resources

The *Declaration of Geneva* primarily addresses the duties of the physician in relation to an individual physician–patient relationship; it focuses on a physician’s primary responsibility for the welfare of the individual patient and ‘does not permit considerations of age, disease or disability, gender …, social standing or any other factor’ to be considered when managing patients (Box 1).^2^

The *Declaration of Geneva* indirectly advocates a ‘first come, first served’ model and assumes the availability of adequate resources to treat all patients. This, however, does not speak to the reality of medical practice, also in high income countries, when the environment and availability of resources are limiting factors for medical professionals who have the intent to give their best but are not able to so because of health system constraints and are often obliged to act as gatekeepers for access to care.

The *Declaration of Geneva* does not recognise triaging (decisions about distribution and utilisation of scarce resources) of patients awaiting investigations and treatment when the burden of disease overwhelms available resources in resource-limited settings. Examples include triaging patients on waiting lists for organ transplantation^4,5^ or selecting patients for ventilatory support during the Coronavirus disease 2019 (COVID-19) pandemic with limited availability of ventilators and intensive care beds.^6^ It also does not permit consideration of factors such as ‘financial toxicity of treatment’ on patients, their families and struggling healthcare systems.^7^

## *Declaration of Geneva* and lower- and middle-income countries

Healthcare services in low- and middle-income countries (LMICs) are (generally) under-resourced, and clinicians and health planners are often faced with overwhelming burdens of disease. They simply cannot treat everyone requiring care. This is the daily lived experience for physicians serving most of the world’s people.

This creates a tension between the ‘first come, first served’ principle implied in the *Declaration of Geneva*, versus deploying limited resources most effectively for the common good. This raises many ethical dilemmas. How does the physician balance the healthcare needs of the individual patient as required by the *Declaration of Geneva*, with that of benefitting other deserving patients, and that of broader society, or ethically ration and prioritise care, and allocate scarce human, physical and financial resources? How does one protect society’s right to access elective care in the face of overwhelming burdens of trauma, emergencies and cancer?^8^ Is it acceptable to ringfence hospital beds, intensive care beds, operating time, funding and staff for elective procedures and care to meet society’s expectation to teach and train well-rounded physicians?^8^ Is it morally acceptable to triage patients based on, for example, prognosis, age, comorbidities, social worth, contributions to society, etc.?

The *Declaration of Geneva* also does not address the physician’s responsibility to allocate scarce resources to benefit broader society as opposed to the individual, for example, weighing up low-cost cataract surgery versus high-cost targeted immunotherapy. With 81 million people driven into financial catastrophe every year simply by costs of getting surgery,^9^ the *Declaration of Geneva* also does not speak to the clinician’s responsibility for the financial wellbeing of his or her patients and their families, and the clinician’s responsibility to society to protect the financial health of both public and private healthcare systems by rationing expensive care, even if it may be to the detriment of individual patients. It also does not place any responsibility or accountability on decision-makers (managers) or policy makers (governments) who allocate finances and resources to health facilities, while the reality is that many physicians in the front-line are not involved in supply chain or procurement processes at facility level.

## Recommendation

Ethics codes should assist physicians to manage the difficult arena also of rationing and priority setting. The *Declaration of Geneva* provides a guide for clinicians and healthcare planners and regulators for ethical practice, but should be amended to recognise the limitations of physicians who have the intent to give their best but are unable to do so because of health system constraints. It should speak not only of a physician’s duty towards his or her individual patient but also a physician’s duty to broader society. It should provide ethical guidance for physicians in limited resource settings about triaging patients when all patients cannot be treated, protecting access to elective care, ensuring teaching and training of well-rounded physicians, ensuring the financial wellness of patients, their families and healthcare systems, deciding who should have access to free healthcare services in a fee-for-service public health system and ensuring accountability for the health and wellness not only of individual patients but also of healthcare systems.
